# Prevalence of Temporal Lobe Epilepsy (TLE) Subtypes and Response to Resective Surgery in Patients with Presumed TLE Undergoing Limbic and Paralimbic Network Exploration with Stereo-Electrodes

**DOI:** 10.3390/jcm14072184

**Published:** 2025-03-23

**Authors:** Irina Podkorytova, Sasha Alick-Lindstrom, Kan Ding, Ryan Hays, Ghazala Perven

**Affiliations:** Department of Neurology, University of Texas Southwestern Medical Center, 5323 Harry Hines Blvd, Dallas, TX 75390-8508, USA; sasha.alick-lindstrom@utsouthwestern.edu (S.A.-L.); kan.ding@utsouthwestern.edu (K.D.); ryan.hays@utsouthwestern.edu (R.H.); ghazala.perven@utsouthwestern.edu (G.P.)

**Keywords:** epilepsy surgery, strereoelectroencephalography (SEEG), temporal lobe epilepsy

## Abstract

**Background/Objectives:** Temporal lobe epilepsy (TLE) responds well to surgical treatment, although a considerable percentage of patients experience seizure recurrence after resection. Relapse from the contralateral mesial temporal lobe, extratemporal lobe epilepsy mimicking TLE, or temporal plus epilepsy might account for surgical failures. **Methods:** We included patients with a pre-implantation hypothesis suggesting TLE, who underwent stereo-EEG (SEEG) evaluation at our institution and had an individual SEEG exploration paradigm with at least twelve stereo-electrodes placed to sixteen brain regions allowing exploration of limbic and paralimbic networks. We analyzed the prevalence of TLE subtypes based on ictal onset localization with SEEG and response to resective surgery. **Results:** Twenty-four subjects met the inclusion criteria. Seven patients had unilateral mesial temporal epilepsy (UMTE), five had bilateral mesial temporal epilepsy (BMTE), five had unilateral neocortical temporal epilepsy (UNTE), six had temporal-plus epilepsy (TPE), one had extratemporal epilepsy (ETE). The number of patients who underwent destructive surgeries and surgical outcomes are as follows: UMTE—all seven patients, Engel I; BMTE- three out of five, Engel I, III, and IV, respectively; UNTE—three out of five, Engel I; TLE mimic (ETE)—one, Engel I; TPE—all six patients, Engel I–three, Engel III–two, Engel IV—one. **Conclusions:** In our study, UMTE was the most frequent TLE subtype (29%), and all patients proceeded to resective surgery with good outcomes. TPE comprised a substantial component (25%) of this cohort with initially presumed TLE, who had a notable proportion of unfavorable outcomes. Larger studies are needed to create guidelines for rational counseling of patients with presumed TLE regarding surgical outcomes.

## 1. Introduction

Temporal lobe epilepsy (TLE) is the most frequent type of epilepsy that is referred to the epilepsy surgical centers, and it usually responds well to surgical treatment [1].

In patients with presumed TLE the use of invasive monitoring is not necessary when semiology and electrophysiologic studies are concordant with a brain lesion (mesial temporal sclerosis (MTS), as an example) that fits the initial localization hypothesis [2].

However, a considerable percentage of TLE patients experience seizure relapse after resection, and defining the epileptogenic zone (EZ) in patients with presumed TLE can be challenging [3,4]. 

Relapses on the contralateral mesial temporal lobe, extratemporal lobe epilepsy mimicking TLE, or temporal plus epilepsy could be among the reasons explaining the surgical failures [5].

Invasive monitoring with stereo-electroencephalography (SEEG) permits the sampling of brain regions, addressing most of the common reasons for surgical failure and allowing for more accurate identification of EZ(s), although the volume of sampled brain tissue is limited [2,6,7,8].

To further investigate the prevalence of TLE subtypes in patients with presumed TLE who underwent SEEG evaluation, we studied 24 patients with one of the pre-implantation hypotheses suggesting temporal lobe epilepsy. These patients underwent SEEG evaluation at our institution and had 12 stereo-electrodes placed in 16 brain regions commonly targeted at our center in patients suspected to have TLE in order to explore the limbic and paralimbic network. Sampling by mesial contacts included the anterior (AH) and posterior hippocampus (PH), amygdala (A), entorhinal cortex (EC), fusiform gyrus (FG), anterior (AI) and posterior insula (PI), orbitofrontal cortex (OC), anterior (AC) and posterior cingulate (PC), precuneus (P), and contralateral hippocampus (CH). Lateral stereo-electrode contacts sampled the temporal (LT), frontal (LF), parietal (LP), and contralateral temporal (CT) neocortex (Table 1).

SEEG localization of the EZ(s) was analyzed to evaluate the prevalence of TLE subtypes and subsequent surgical outcomes to add to the limited body of literature on the topic.

## 2. Materials and Methods

### 2.1. Patients

This is a retrospective, observational study conducted after institutional review board approval. A total of 200 consecutive patients with drug-resistant focal epilepsy who underwent invasive pre-surgical evaluation with stereo-electrodes at our center were screened. We included patients with one of the pre-implantation hypotheses suggesting TLE, who underwent SEEG evaluation at our institution and had 12 stereo-electrodes placed in the 16 brain regions (Table 1); additional stereo-electrodes could be placed according to the hypotheses. Patients who underwent previous temporal lobectomy or subdural grid evaluations were excluded from our cohort. Twenty-four patients met the inclusion criteria.

All 24 patients underwent a comprehensive workup including history and neurologic exam, scalp video EEG monitoring, 3 Tesla MRI brain, functional MRI, and neuropsychological testing, after which the data were insufficient to conclusively identify EZ, so SEEG was pursued as part of routine clinical care. The recommendations to proceed with SEEG evaluation and SEEG implantation maps were discussed by a multidisciplinary committee, including at least three board-certified epileptologists, a neurosurgeon, a neuroradiologist, and the neuropsychologists.

### 2.2. MRI Protocol

All MRI scans were performed on 3 Tesla MRI scanners. The brain MRI protocol for epilepsy patients included the following non-contrast high-resolution sequences: two-dimensional axial diffusion-weighted imaging (2D AX DWI) and T2-weighted fluid-attenuated inversion recovery turbo-spin echo (2D AX T2W FLAIR TSE), coronal oblique 2D COR T2W TSE and 2D COR T2W FLAIR TSE, and three-dimensional axial susceptibility weighted imaging fast field echo/T2 gradient echo (3D AX SWI FFE/T2 GRE) and sagittal T1-weighted magnetization prepared rapid gradient echo/isotropic turbo field echo (3D SAG T1W MPRAGE/TFE ISO).

### 2.3. Sixteen Brain Regions Sampled with Stereo-Electrodes

All 24 patients in our study had sampling of 16 brain regions by 12 stereo-electrodes (additional stereo-electrodes could be placed according to the hypothesis); mesial contacts were placed to anterior and posterior hippocampus, amygdala, entorhinal cortex, fusiform gyrus, anterior and posterior insula, orbitofrontal cortex, anterior and posterior cingulate, precuneus, and contralateral hippocampus; lateral SE contacts targeted temporal, frontal, parietal and contralateral temporal neocortex.

### 2.4. SEEG Evaluation

SEEG implantation was performed using established methods [2,9]. Trajectory plans were performed using stereotactic planning software from the ROSA system (Zimmer Biomet Robotics, Montpellier, France). Postoperatively, patients were observed in the epilepsy monitoring unit, and a postoperative CT scan was obtained and co-registered to the preoperative MRI to allow for localization of each electrode and electrode contact. At least two experts visually reviewed 100% of the neurophysiologic data obtained from SEEG and agreed on the localization of ictal onset(s).

### 2.5. Surgical Treatment

Surgical interventions were planned based on the results of the SEEG analysis in accordance with multidisciplinary epilepsy conference recommendations. Surgical options included destructive surgery (open surgical resection or laser interstitial thermal therapy (LITT)), responsive neurostimulation (RNS, manufacturer—Neuropace, Mountain View, CA, USA) placement, or a combination of destructive surgery and RNS.

The open surgical resections were a standard anterior temporal lobectomy, which included resection of the hippocampus or neocortex resection based on SEEG localization results. The standard anterior temporal lobectomy involved the removal of the anterior temporal neocortex, the parahippocampal gyrus, the amygdala, and the hippocampus with the goal of removing as much of the hippocampus as possible, the uncus, and surrounding structures. The extension of the temporal neocortex resection was 4.5 cm posteriorly to the temporal tip on the language-dominant side and 5.5 cm on the non-dominant side.

### 2.6. Surgical Outcomes

Surgical outcomes were classified according to the Engel classification system [10] and were based on patient interviews at the time of the last clinic or telephone follow-up.

### 2.7. Statistics

Statistical analyses were performed using SPSS 20 software (IBM Co., Armonk, NY, USA). The statistical methods were descriptive statistics; group differences were compared using Fisher’s exact test. Significance was defined as a probability (*p*) value < 0.05.

## 3. Results

### 3.1. Patients

Clinical, imaging, and electrophysiologic data are summarized in Table 2. Of the 24 included patients, 12 were male and 12 were female. The mean age of seizure onset was 23.5 years (range 5 months–45 years), and the average age at the time of SEEG was 34.5 years (range 18–61 years). The mean duration of epilepsy at the time of SEEG implantation was 10.8 years (range 3–30 years). All patients had drug-resistant epilepsy (mean number of antiseizure medications (ASM) at the time of SEEG 2.6, range 2–4) as defined by the International League Against Epilepsy [11].

### 3.2. MRI

Twelve out of twenty-four patients had normal brain MRI, and 12 had MRI lesion(s). Five out of twenty-four patients had an MRI lesion related to the SEEG ictal onset (one—mesial, three—lateral temporal, and one—extratemporal). In two out of five patients, an MRI lesion related to the SEEG ictal onset was found after stereo-electrodes placement. Nine patients have brain lesion(s) not related to ictal onset (three—temporal, three—extratemporal, three—temporal and extratemporal). MRI findings are summarized in Table 2.

### 3.3. Reasons to Recommend SEEG Evaluations

The reasons for undergoing invasive monitoring with SEEG are summarized in Table 2 and include non-lesional MRI [12,13], MRI lesion outside of the temporal lobe [5], semiology required additional coverage [14,15], discordant non-invasive evaluation results [2], or combination of these factors.

### 3.4. SEEG Ictal Onset(s)

Table 3 listed SEEG ictal onset localization for each patient. More than one of 16 investigated regions could be involved in the ictal onset of the same seizure in 10 out of 24 patients.

Thirteen patients had involvement of the unilateral anterior hippocampus in the ictal onset; six, the posterior hippocampus; three, the amygdala; four, the entorhinal cortex; one, the fusiform gyrus; one, the anterior insula; three, the posterior insula; one, the orbitofrontal cortex; ten, the lateral temporal; three, the lateral frontal; and six, the contralateral hippocampus.

Seven out of twenty-four patients had unilateral mesial temporal onset (which could include AH, PH, A, EC), five—bilateral mesial temporal (AH, PH, A, EC, CH), four—unilateral LT, one—unilateral mesial and lateral temporal (AH, A, EC, LT), two—unilateral mesial and lateral temporal, and extratemporal (AH, PH, LT, LF, AI), one—unilateral extratemporal (LF), three—unilateral lateral temporal and extratemporal (LT, PI, LF, OC), and one—bilateral mesial and lateral temporal and extratemporal (LT, PI, CH), Table 4.

The patient who had simultaneous unilateral mesial and lateral temporal ictal onset was diagnosed with temporal pole encephalocele after SEEG. The peri-lesional area was not sampled with stereo-electrodes. Therefore, the observed SEEG ictal onset represented the spread of the ictal activity. This patient was included in the unilateral neocortical temporal lobe epilepsy subgroup in this study.

**Figure 1:**
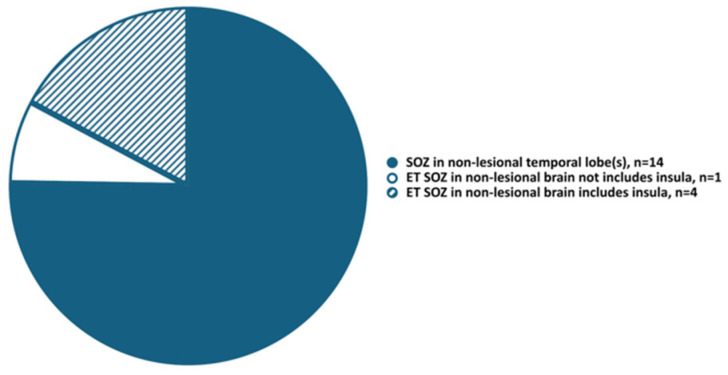
Seizure onset zone in non-lesional brain areas. ET, extratemporal; SOZ, seizure onset zone.

In patients with presumed TLE and SEEG ictal onset non-related to MRI lesion, the insula was an extratemporal structure most frequently involved in EZ (four out of five patients, *p* = 0.001, Figure 1), and the posterior insula was more frequently involved in ictal onset than anterior insula (in three and one patients respectively, Table 4).

### 3.5. Prevalence of TLE Subtypes

**Figure 2:**
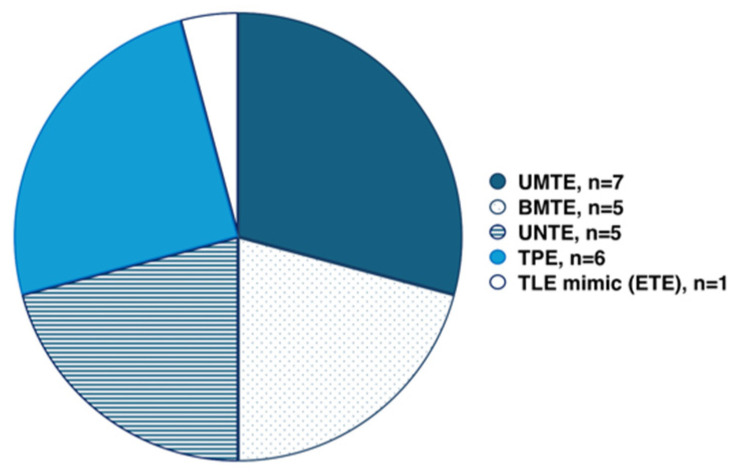
Temporal lobe epilepsy subtypes. BMTE, bilateral mesial temporal lobe epilepsy; ETE, extratemporal epilepsy; TLE, temporal lobe epilepsy; UMTE, unilateral mesial temporal lobe epilepsy; UNTE, unilateral neocortical temporal lobe epilepsy.

Based on the ictal onset localized with SEEG, seven patients (29%) had unilateral mesial temporal epilepsy (UMTE), five (21%)—bilateral mesial temporal epilepsy (BMTE), five (21%)—unilateral neocortical temporal epilepsy (UNTE), six (25%)—temporal-plus epilepsy (TPE), and one (4%)—extratemporal epilepsy (ETE), Figure 2.

### 3.6. Surgical Outcomes

Surgical outcomes per TLE subtype are summarized in Table 5. Table 3 lists the surgical approach and outcome for each patient. Therapeutic surgery was completed on average 3 months after SEEG evaluation (range 1.5–6.5 months) for all patients except one who had left ATL two years after SEEG. Nineteen patients had resective surgery only (mean follow-up 56 (16–89) months), two patients underwent RNS placement (45 and 60-month follow-up), then one of these patients had LITT of the left hippocampus (48-month follow-up post-LITT). One patient underwent a partial lesion resection combined with RNS as one surgical procedure (81-month follow-up), and one patient had more than one post-SEEG surgery (Engel III after both resections and RNS placement, 69-month follow-up after the first resection). In total, after destructive surgery, 15 patients were Engel I, three patients were Engel III, and two patients were Engel IV. In the Engel I patient subgroup, 9 out of 15 patients were completely seizure-free after surgery (Engel IA); 4 out of 9 patients had UMTE, two had UNTE, and one patient each had BMTE, TPE, and ETE. In the TPE patient subgroup with Engel class III and IV outcomes, the ictal onset included unilateral dorsal frontal, orbito-frontal (encephalomalacia), and lateral temporal cortex in one patient, unilateral non-lesional temporal neocortex and posterior insula in the second, and unilateral non-lesional anterior hippocampus, temporal neocortex and anterior insula in the third patient. In the BMTE patient subgroup, unfavorable outcomes were observed after left hippocampus LITT and left temporal lobectomy (Engel class III and IV, respectively).

#### 3.6.1. Unilateral Mesial Temporal Lobe Epilepsy

In our patient cohort, the most frequent EZ localization (seven out of twenty-four) were unilateral mesial temporal structures, including AH (three—left, one—right), AH and PH (one—left), and AH, PH, EC, A simultaneously (one—left, one—right). All these patients did not have mesial temporal lobe lesion(s) related to EZ, two out of seven had lesion(s) non-related to EZ: one patient had extratemporal lesion unilateral to the EZ (frontal horn periventricular nodular heterotopia), and one patient had temporal neocortical and extratemporal lesions (encephalomalacia) ipsilateral to EZ. All seven patients underwent a standard anterior temporal lobectomy (SATL) and had an Engel I outcome with an average follow-up duration of 52 months (range 20–89). The surgical pathology showed MTS in one patient and was normal in six patients.

#### 3.6.2. Bilateral Mesial Temporal Lobe Epilepsy

Five out of twenty-four patients in our cohort had bilateral mesial temporal ictal onsets. Four out of five did not have MRI lesion(s) related to EZ, and one (Patient 9) reportedly had a loss of gray-white matter differentiation of the left anteromesial temporal lobe. There were no MRI lesions outside of the mesial temporal lobes.

Three out of five patients had a bilateral temporal pre-implantation hypothesis. During SEEG evaluation, Patient 9 (with unilateral mesial temporal lobe MRI lesion) had clinical seizures from the left and right hippocampus; bilateral mesial temporal RNS leads were placed and are currently recording seizures from both hippocampi. Patient 13 had clinical seizures with the broad ictal onset over right and left mesial temporal structures (right entorhinal cortex, anterior and posterior hippocampus, and left anterior and posterior hippocampus and amygdala), but initial negativity was consistently seen over the electrode contacts in the right entorhinal cortex. This patient also had sub-clinical seizures from the left hippocampus. Given bilateral independent mesial temporal ictal activity captured during SEEG evaluation and the broad ictal onset of clinical seizures, bilateral temporal RNS lead placement was recommended, and surgery is pending. Patient 20 had clinical seizures starting from the left hippocampus and sub-clinical seizures starting from both hippocampi. Left temporal lobectomy was completed 2 years after SEEG with an Engel class IV outcome (38-month follow-up).

Two out of five patients had a unilateral temporal pre-implantation hypothesis, but during SEEG, focal unaware seizures were recorded from one mesial temporal lobe, and Patient 2 had autonomic auras from CH; Patient 17 had sub-clinical seizures from CH off ASM. Patient 2 had RNS in the bilateral hippocampi for 42 months and had almost the same seizure frequency as preoperatively. Also, during the last two years, she had seizures starting from the left hippocampus only; therefore, her RNS was explanted, and the patient proceeded to left hippocampus LITT with an Engel class III outcome (46-month follow-up). Patient 17 underwent right SATL and is currently 60 months seizure-free on ASM.

#### 3.6.3. Unilateral Temporal Neocortical Epilepsy

Four out of twenty-four patients in our cohort had unilateral neocortical temporal ictal onsets. Three out of four patients did not have MRI lesions related to SEEG ictal onset; one (Patient 7) had a left fusiform gyrus lesion, and seizures started from the peri-lesional area. Isolated ictal onset from FG was related to FG lesion only in our patient cohort.

In the “lateral temporal” sub-group of patients who did not have a lesion related to SEEG ictal onset, Patient 3 had normal PET, very focal SEEG ictal onset of his clinical seizures within the middle part of the right superior temporal sulcus, and currently is seizure free during 85 months after the right temporal neocortex resection (normal pathology) on ASM, although he had two seizures after his surgery (four and twelve-months post-op) while decreasing ASM.

Patient 11 had left temporal and adjacent parietal cortex PET hypometabolism, a broad left temporal ictal onset of his clinical seizures with early involvement of adjacent parietal cortex, and sub-clinical seizures from the left temporal operculum, pending RNS placement.

Patient 15 had right temporal and right occipital lobe PET hypometabolism; his SEEG clinical and sub-clinical seizures were focally localized to the middle part of the right middle temporal gyrus. He had bilateral MTS non-related to his EZ and was seizure-free during 54 months on ASM after the right SATL. His surgical pathology showed MTS in the resected hippocampus, but no abnormalities were found in the resected neocortex.

Patient 1 had broad unilateral mesial and lateral temporal ictal onset recorded with SEEG but was included in the UNTE patient subgroup. This patient underwent two SEEG evaluations; the first implantation was aborted after six electrode (mesial contacts sampling AH, PH, A, TP, parahippocampal gyrus, PC) placements due to suspected intracranial hemorrhage, which was not confirmed with images. The broad ictal onset involving mesial and lateral temporal structures was recorded; therefore, the second SEEG was recommended, which included all electrodes from the TLE exploration paradigm, but the recorded ictal onset was the same, simultaneously involving AH, A, EC, and LT cortices. The preoperative brain images were reportedly non-lesional, but during SEEG evaluation, images were re-reviewed, and the temporal pole encephalocele was noted; the patient underwent left temporal pole resections and is 16 months seizure-free despite broad ictal onset recorded during two SEEG evaluations.

#### 3.6.4. Isolated Extratemporal Epilepsy

Patient 22 had discordant pre-surgical evaluation results; therefore, SEEG was recommended. She had an isolated extratemporal SEEG ictal onset seen in the right superior frontal sulcus and middle frontal gyrus followed by fast spread to the ipsilateral temporal neocortex and currently is seizure-free for 60 months after the right frontal cortex resection (normal pathology).

#### 3.6.5. Temporal Plus Epilepsy

Six out of twenty-four patients had different combinations of temporal and extratemporal ictal onsets (Patients 10, 12, 14, 16, 18, and 24).

We found that in patients with presumed TLE and EZ non-related to MRI lesions, the insula is the most frequently involved in EZ extratemporal structure (four out of five patients, *p* = 0.001), the posterior insula was more frequently involved in ictal onset than anterior insula (in three and one patients respectively, Patients 16, 18, 24, and Patient 14), and involvement of the insula in EZ could be linked to unfavorable epilepsy surgery outcomes (two out of four patients: Patient 14—anterior insula, not resected due to risk of complications, and Patient 16—posterior insula, resected).

## 4. Discussion

We presented a study that demonstrated the prevalence of TLE subtypes and the value of SEEG exploration to define the TLE subtype. We demonstrated that unilateral mesial temporal lobe epilepsy was the most frequent TLE subtype, and patients with unilateral mesial temporal lobe epilepsy have the best destructive epilepsy surgery outcome despite the absence of MRI lesions related to EZ. Temporal plus epilepsy was the next by prevalence in our TLE patient cohort, and a significant proportion of patients with temporal plus epilepsy had unfavorable epilepsy surgery outcomes. Bilateral mesial temporal lobe epilepsy and unilateral neocortical temporal lobe epilepsy were equally represented in our patient cohort, and one patient had extratemporal lobe epilepsy mimicking TLE.

We found that in patients with presumed TLE and EZ non-related to MRI lesions, the insula is the most frequently involved in EZ extratemporal structure (four out of five patients, *p* = 0.001), the posterior insula was more frequently involved in ictal onset than the anterior insula (in three and one patients, respectively), and involvement of the insula in EZ indicated the presence of temporal plus epilepsy and could be linked to unfavorable epilepsy surgery outcomes (two out of four patients: one—anterior, and one—posterior insula). These findings are concordant with previous reports that the insula may be an important cause of surgical failure in patients with TLE [16,17,18,19].

We replicated data from previous studies suggesting that temporal and temporal plus epilepsies could be difficult to differentiate based on general clinical features or MRI data only [20].

Although orbito-frontal cortex epilepsy was reported in the literature as the frequent TLE mimic [21,22,23,24], and involvement of the orbitofrontal cortex in the epileptogenic network was linked to less favorable resective surgery outcomes in a study performed on SDE patients without insula sampling [25], in our cohort, the orbitofrontal cortex was involved in ictal onset only in one patient with temporal plus epilepsy (Patient 12), and it was related to encephalomalacia in this area (Engel class III outcome after resection, pathology-remote infarct).

The only patient with an isolated extratemporal epileptogenic zone in our cohort had epileptogenicity arising from the non-eloquent, non-lesional dorsal frontal cortex, and our TLE stereo-electrodes placement paradigm was instrumental in localizing EZ in this patient with seizure semiology mimicking TLE [5].

Seizure relapse from the temporal lobe contralateral to the side of surgery is an important cause of failure in the surgical treatment of TLE, estimated to represent 12–30% of patients who fail surgery [26,27,28,29,30].

We previously published a study reporting 4 out of 28 patients with unilateral temporal scalp EEG seizures who had bilateral independent mesial temporal SEEG seizures, although all non-invasive evaluation data suggested a unilateral temporal lobe EZ [13]. Therefore, we propose that patients with presumed TLE would benefit from bilateral invasive evaluation of mesial temporal structures with SE to predict those patients who would be at the most risk for surgical failure and neuropsychological worsening [31].

Several studies have aimed to identify clinical, electrographic, and imaging differences between mesial and lateral TLE, but this distinction can be challenging as some features said to be typical of lateral TLE can also be found in mesial TLE [32,33,34,35,36,37]. Also, some patients show variable implications of both the mesial and lateral parts of the temporal lobe in seizure onset [28,38].

From our center experience, the patients with presumed temporal neocortical epilepsy need mesial temporal structures (hippocampus, amygdala, and entorhinal cortex) sampling with SE because the ictal onset could be broad or multifocal involving lateral and mesial temporal strictures in different combinations, including not only seizures starting from the temporal neocortex and hippocampus but in some patients, seizures starting from the lateral temporal cortex and from the amygdala (not involving hippocampus at the onset), what would affect the surgical decision to recommend temporal neocortex resection vs temporal lobectomy including mesial temporal structures. Amygdala and entorhinal cortex sampling are not always included in SEEG paradigm for TLE exploration across epilepsy centers. We think that undiagnosed involvement of these structures in EZ, which is otherwise localized to the temporal neocortical, could be an explanation for better resective surgery outcomes in patients with presumable neocortical TLE after temporal lobectomy including mesial temporal structures, compared to temporal neocortex resection only [39].

Seizure freedom after non-lesional temporal neocortex resection in two individuals from our sub-group of patients with unilateral temporal neocortical ictal onset requires further understanding of the underlying mechanism of their epilepsy. Also, their ASM was not discontinued after resection.

One patient (Patient 1) with broad unilateral mesial and lateral temporal SEEG ictal onset had a temporal pole encephalocele revealed after stereo-electrodes placement, and this patient is seizure-free after the temporal pole resection. We think this patient could have temporal neocortical lesion-related ictal onset, which was not localized due to stereo-electrodes sampling bias since ictal onset cannot be observed from unsampled areas, and broad onset may represent spread from a more isolated focus.

SEEG evaluation results and surgery outcomes of Patients 1 and 10 suggested that seizure freedom is possible despite broad SEEG ictal onset(s) if EZ is related to an epileptogenic lesion that is resected.

Identification of a structural lesion on MRI is associated with favorable seizure outcomes after surgery [40]; therefore, it is critical to integrate complex imaging techniques into routine clinical practice [41,42,43].

Nevertheless, in our patient cohort, only five out of fourteen MRI lesions were associated with EZ (Table 2 and Table 3). Our findings are consistent with previous studies suggesting that MRI structural lesions, including presumed highly epileptogenic such as MTS, are not necessarily related to EZ(s). Therefore, alternative epilepsy etiologies and implantation strategies should be considered prior to stereo-electrodes placement [28,44,45,46,47].

Our study has several limitations. Most significantly, the retrospective design and small sample size limit our ability to make definitive conclusions. Rather, we offer a description of our observations and how this might affect practice. Ultimately, larger, likely multicenter, prospective studies are needed since sample size limits studies reporting TLE subgroups’ prevalence and surgical outcomes. Additionally, several of our patients had relatively short follow-up times, limiting our ability to analyze the accuracy of EZ localization and the long-term efficacy of subsequent surgical treatment. Moreover, ictal onset was complex at times, and a precise ictal onset was difficult to definitely determine. Sampling bias contributes further to this problem since ictal onset cannot be observed from unsampled areas, and broad onset may represent spread from a more isolated focus.

## 5. Conclusions

Our study demonstrates that unilateral mesial temporal lobe epilepsy was the most frequent TLE subtype (29%), and all patients proceeded to resective surgery with good outcomes despite the absence of MRI lesions related to EZ. Temporal plus epilepsy comprised a substantial component (25%) of this cohort with initially (pre-SEEG) presumed TLE, and a significant proportion of patients with temporal plus epilepsy had unfavorable epilepsy surgery outcomes. Although our patient number limits definitive conclusions, we observed a trend suggesting that in patients with EZ related to non-lesional MRI, the insula is an extratemporal structure most frequently involved in ictal onset in patients with pre-implantation hypotheses suggesting temporal lobe seizures, and involvement of insula in ictal onset in patients with presumed TLE could be related to unfavorable resective surgery outcomes. Ultimately, larger studies are needed to replicate these findings and to create guidelines for effective exploration of medically refractory TLE with SEEG in order to further select optimal surgical treatment strategies and to improve surgical outcomes and quality of life in patients with drug-resistant focal epilepsy.

The other area for further investigation could be a goal to create guidelines for rational counseling of patients with presumed TLE regarding surgical outcomes and overall to improve the reliability of prognosis regarding epilepsy surgery results in this patient cohort.

## Figures and Tables

**Table 1:** Sixteen brain regions sampled with 12 stereo-electrodes.

	Mesial Contacts	Lateral Contacts
1	Anterior hippocampus (AH)	Lateral temporal (LT), including middle (MTG), inferior (ITG), or superior (STG) temporal gyrus
2	Posterior hippocampus (PH)	LT (posterior part of MTG vs. ITG vs. STG)
3	Entorhinal cortex (EC)	LT (temporal pole (TP) vs. anterior part of MTG vs. ITG vs. STG)
4	Amygdala (A)	LT (TP vs. anterior part of MTG vs. ITG vs. STG)
5	Posterior part of the fusiform gyrus (FG)	LT (posterior part of MTG vs. ITG)
6	Anterior insula (AI)	Lateral frontal (LF, including Superior frontal gyrus (SFG) vs. Middle frontal gyrus (MFG)
7	Posterior insula (PI)	LT (middle part of STG)
8	Orbitofrontal cortex (OC)	LF (Inferior frontal gyrus (IFG))
9	Anterior cingulate (AC)	LF (IFG vs. MFG)
10	Posterior cingulate (PC)	Lateral parietal (LP)
11	Precuneus (P)	LP
12	Contralateral hippocampus (CH)	LT (contralateral MTG vs. STG vs. ITG)

**Table 2:** Summary of patient characteristics and pre-surgical workup.

P A T I E N T	Age at SEEG/Gender/Handedness	Epilepsy Onset Age/Duration by SEEG (Years)	Seizure Semiology	Scalp EEG-Ictal Clinical/Ictal Sub Clinical/Interictal SW	MRI All Findings *	PET	Language Dominance, fMRI/Wada	Neuro Psychology Deficit	Ictal SPECT/MEG	Reason for SEEG
1	45/Fe/R	40/5	Aura (psychic vs non-specific) => Dyscognitive => +/− R head turn => FBTC	Lateralized L/none/LT	**Left temporal pole encephalocele (identified after SEEG)**; small left hippocampus cyst	Normal	Artifact-language not localized/Left	Non-lateralized, non-localized	n/a/n/a	Non-lesional MRI
2	39/Fe/R	36/3	Aura (autonomic/L arm sensory) => dyscognitive/oral automatisms (+/−RINCH)	LAT/none/LT	None	Normal	Left/n/a	LF	n/a/LT, anterior mesial and lateral	Non-lesional MRI; semiology required additional coverage; discordant non-invasive evaluation results
3	21/M/R	13/8	Autonomic aura (nausea) => left head and mouth clonic/left shoulder clonic	RT/none/RT-rare	None	Normal	n/a n/a	Non-lateralized, non-localized	Symmetric/n/a	Non-lesional MRI; discordant non-invasive evaluation results
4	22/M/R	19/3	Aura (lightheadedness) => dyscognitive => R hand dystonic posturing w/right facial clonic movement	LT/none/L TIRDA	None	Normal	Left/n/a	Bi F	LT/n/a	Non-lesional MRI; semiology required additional coverage; discordant non-invasive evaluation results
5	33/Fe/L	29/4	1. Aura (left frontal HA or aphasia) => dyscognitive/automotor. 2. R versive head turn => FBTC	LT/LT/LT-rare	Left periventricular frontal heterotopia (anterior horn of LV)	Normal	Bilateral, LEFT dominance/n/a	R FT	n/a/n/a	MRI lesion outside of the temporal lobe; semiology required additional coverage; discordant non-invasive evaluation results
6	61/M/R	45/16	Dyscognitive => bimanual automatisms	Onset obscured, spread RT none/RAT (frequent), LT, BF (rare)	None	Normal	Left/n/a	Non-lateralized, non-localized	RT/n/a	Non-lesional MRI; discordant non-invasive evaluation results
7	21/Fe/R	13/8	Aura (non-specific) => Dyscognitive/gestural automatisms	LT (after clinical onset)/none/LT, L TIRDA	**Left fusiform gyrus lesion;** questionable FCD in the medial aspect of the left frontal pole, questionable right-sided hippocampal atrophy	n/a	Left/n/a	Bi T	n/a/n/a	MRI lesion outside of the temporal lobe; discordant non-invasive evaluation results
8	26/Fe/R	15/11	1. Dyscognitive/automotor (L hand automatisms, R dystonic). 2. FBTC in sleep	LT/none/LT	None	LT	n/a/left	Left hemispheric	n/a/n/a	Non-lesional MRI; discordant non-invasive evaluation results
9	55/M/R	25/30	1. Automotor => CPS/dyscognitive. 2. FBTC-Rare.	LT-9/10, RT-1/10/none/LT-frequent, RT- rare	**Loss of gray-white differentiation of the anteromesial left temporal lobe**	LT	Left/left	Non-lateralized, non-localized	n/a/n/a	Discordant non-invasive evaluation results
10	45/M/R	30/15	1. Aura (smell, auditory hallucination, loss of train of thought) => gestural automatisms/complex motor => speech arrest. 2. Dyscognitive =>R head turn, FBTC	Non-lateralizing/none/LT	**Left posterior temporal encephalomalacia** (identified during SEEG evaluation)	Normal	Left/n/a	R hemisphere	Symmetric/LT	Non-lesional MRI; semiology required additional coverage; discordant non-invasive evaluation results
11	27/M/L	11/16	Automotor (L automatisms, R dystonic) => R face clonic => R versive head turn => FBTC	LT/None/LT	None	LT, LP	Left/n/a	Non-lateralized, non-localized	RT/n/a	Non-lesional MRI; discordant non-invasive evaluation results
12	25/Fe/R	14/11	1. Aura (dizziness) => dyscognitive, oral automatisms. 2. FBTC (rare, R versive head turn)	Lateralized L hemisphere/none/broad LT, PFA left temporal	**Left frontal encephalomalacia**	LF (lesion)	Left/n/a	L > R hemispheric	n/a/n/a	MRI lesion outside of the temporal lobe; semiology required additional coverage; discordant non-invasive evaluation results
13	22/M/R	19/3	Aura (dejavu, auditory) => dyscognitive/automotor (R foot tapping)	BT (FA-RT evolution, FU-LT evolution)/None/Rare L FT	None	Normal	Left/n/a	L hemispheric	n/a/n/a	Non-lesional MRI; semiology required additional coverage; discordant non-invasive evaluation results
14	43/M/R	36/7	1. Abrupt FBTC. 2. Dyscognitive, oral automatisms => excessive salivation and tearing	RT, very rapid spread to LT/none/RT >> LT	None	RT	Left/n/a	RT	n/a/n/a	Non-lesional MRI; semiology required additional coverage; discordant non-invasive evaluation results
15	25/M/R	0.5/25	1. Autonomic (heavy breathing, excessive sweating) => dyscognitive and oral automatisms. 2. FBTC	R FC/RT/RT	R MTS, bilateral hippocampi atrophy, left parietal FCD	RT, RO	Left/n/a	Non-lateralized, non-localized	n/a/n/a	MRI lesion outside of the temporal lobe; semiology required additional coverage; discordant non-invasive evaluation results
16	18/M/R	8 y/10 y	Aura (chills- non-specific) => dyscognitive => left face tonic, left head turn => FBTC (frequent)	RT, R FT, Broad R hemispheric/None/RT	Questionable right middle temporal gyrus FCD and early right MTS	R FT	Left/n/a	BT	n/a/RT (mesial)	Semiology required additional coverage; discordant non-invasive evaluation results
17	44/M/R	32/12	1. Rare aura (dejavu, autonomic tachycardic/diaphoresis) => dyscognitive and automatisms (R hand pounding on chest, oral), ictal speech. 2. L versive head turn =>FBTC (rare)	RT posterior/None/RT anterior	None	Normal	Left/n/a	RT (mesial)	n/a/n/a	Non-lesional MRI; semiology required additional coverage; discordant non-invasive evaluation results
18	43/M/A	38/6	Dyscognitive => left hand clonic/dystonic	RT/None/RT	Bullet fragment in skull right parietal, no brain damage	Normal	Left/n/a	LT	n/a/n/a	MRI lesion outside of the temporal lobe; discordant non-invasive evaluation results
19	57/Fe/R	52/5	Dyscognitive -=> R hand automatism => R versive head turn => FBTC	Non-localizing onset, LT evolution/None/RT, LT	None	LT	Left/n/a	Non-lateralized, non-localized	n/a/n/a	Non-lesional MRI; discordant non-invasive evaluation results
20	30/Fe/R	20/10	Aura (autonomic-HV, diaphoresis, dry mouth) => dyscognitive, bimanual automatisms => FBTC (right head turn)	L FT/LT/Bi FT	None	Normal	Co-dominant/n/a	Dominant hemispheric	n/a/n/a	Non-lesional MRI; semiology required additional coverage; discordant non-invasive evaluation results
21	23/Fe/R	15/8	Aura (non-specific tingling all over body) => R face tonic/dyscognitive => FBTC	LT/None/LT	None	LT	Bilateral, slight LEFT dominance/n/a	Dominant temporal lobe	n/a/Normal	Non-lesional MRI; semiology required additional coverage; discordant non-invasive evaluation results
22	48/Fe/L	23/25	1. Myoclonic. 2. Aura (autonomic, difficulty breathing) => dyscognitive, clicking sounds => L head version and FBTC. 3. Aura (Sensory, nausea, head pain, anxiety, visual disturbance) => dyscognitive	RF/None/RF	Subtle increased cortical thickness and increased T2 signal in right calcarine cortex	RT (mesial)	Left/n/a	Non-lateralized, non-localized	n/a/n/a	MRI lesion outside of the temporal lobe; semiology required additional coverage; discordant non-invasive evaluation results
23	26/Fe/R	16/10	Aura (dejavu, whole body tingling) => dyscognitive, oral automatisms => FBTC (R versive head turn)	LT/LT/LT, RT	Small areas of encephalomalacia involving the left middle, inferior frontal gyri, left superior, middle temporal gyri	Bi T (mesial	Left/n/a	L hemispheric, LT (mesial)	RT, RF/LF, LT perilesional	MRI lesion outside of the temporal lobe; semiology required additional coverage; discordant non-invasive evaluation results
24	24/Fe/R	15/9	Aura (feels blind, deaf and mute) => dyscognitive, bimanual automatisms, R facial twitching/head jerking	L FT/LT/LT	Early left MTS	LT	Bilateral, LEFT dominance/n/a	Non-lateralized, non-localized	n/a/n/a	Semiology required additional coverage; discordant non-invasive evaluation results

Bi, bilateral; F, frontal; FCD, focal cortical dysplasia; Fe, female; FBTC, focal to bilateral tonic-clonic; fMRI, functional MRI; L, left; M, male; MEG, magnetoencephalography; MTS, mesial temporal sclerosis; PFA, paroxysmal fast activity; PET, positron emission tomography; P, parietal; R, right; RINCH, rhythmic ictal non-clonic hand movements; SEEG, stereoelectroencephalography; SPECT, single-photon emission computerized tomography; SW, sharp waves; T, temporal; TIRDA, temporal intermittent rhythmic delta activity. * MRI lesions related to SEEG ictal onset are highlighted in bold and underscored.

**Table 3:** SEEG ictal onset localization, post-SEEG surgery, and outcome.

P A T I E N T	TLE Subtype	SEEG Ictal Onset-Clinical Seizures	SEEG Ictal Onset-Sub-Clinical Seizures	MRI-Epilepsy Etiology, Description/ Classification	Post-SEEG Surgery	Surgical Pathology	Outcome, Engel Class/Follow-Up Duration, Months
1	UNTE	AH, A, EC, LT	None	Anterior temporal pole encephalocele (identified after SEEG)/UNTL	Ltemporal neocortex resection	Normal	IA/16
2	BMTE	1. AH, PH, A, EC; 2. CH	None	Non-lesional	RNS bilateral hippocampi, then L hippocampus LITT	N/A	IIIA/46
3	UNTE	LT	None	Non-lesional	R temporal neocortex resection	Normal	IC/85
4	UMTE	AH	None	Non-lesional	L standard ATL	MTS	IC/89
5	UMTE	AH	None	Non-lesional	L standard ATL	Normal	IA/31
6	UMTE	AH	None	Non-lesional	R standard ATL	Normal	IA/20
7	UNTE	FG	None	Left Fusiform gyrus lesion/UNTL	R partial lesionectomy + RNS	Normal	N/A
8	UMTE	AH, PH, A, EC	None	Non-lesional	R standard ATL	Normal	IA/69
9	BMTE	1. AH; 2. CH	None	Loss of gray-white differentiation of the anteromesial left temporal lobe/UMTL	RNS bilateral hippocampi	N/A	N/A
10	TPE	1. broad LT; 2. LF; 3. AH, PH	None	Left posterior lateral temporal post-traumatic encephalomalacia (found on MRI re-review during SEEG)/UNTL	L mesial temporal and anterior and posterior lateral temporal) + L SFS resection + LF MST	Left posterior temporal-remote infarcts, left hippocampus, amygdala-normal	ID/56 mo
11	UNTE	LT	Left temporal operculum	Normal	Pending	N/A	N/A
12	TPE	1. Broad LF+OC; 2. Broad LT (when in cluster)	None	Left frontal encephalomalacia/ETL	L orbito-frontal/ lateral frontal lesion resection	Remote infarct	III/87 mo
13	BMTE	EC	Contralateral hippocampus	Normal	Pending	N/A	N/A
14	TPE	AH, LT, AI	None	Normal	R standard ATL	Normal	IVB/74
15	UNTE	RT	Unilateral (Rrght) neocortical temporal lobe	Normal	R standard ATL	MTS	IA/54
16	TPE	1. LT, 2. PI	None	Normal	1. R posterior temporal/posterior insula resection; 2. R standard ATL + insula resection (part of anterior insula not resected); 3. RNS R frontal operculum and R temporal resection edge	Normal	IIIA/69
17	BMTE	AH	Contralateral hippocampus (left)	Normal	R standard ATL	Normal	IA/60
18	TPE	LT, PI	Contralateral hippocampus (left), unilateral posterior insula (right)	Normal	R standard ATL	Normal	IB/61
19	UMTE	AH, PH, A, EC	Hippocampus (left)	Normal	L standard ATL	Normal	ID/57
20	BMTE	AH, PH	1. Right hippocampus, 2. Left hippocampus	Normal	L standard ATL	N/A	IV/38
21	UMTE	AH, PH	Unilateral (left) mesial temporal lobe	Normal	L standard ATL	Normal	IA/39
22	ETE	LF	Unilateral (right) extratemporal (frontal)	Normal	R frontal lobectomy	Normal	IA/60
23	UMTE	AH	None	Normal	L standard ATL	Normal	ID/60
24	TPE	1. PI; 2. LT, PI	Unilateral (left) extratemporal (insula) and lateral temporal	Normal	L temporal operculum and posterior insula resection	N/A	IA/50

A, amygdala; AH, anterior hippocampus; AI, anterior insula; ATL, anterior temporal lobectomy; BMTE, bilateral mesial temporal epilepsy; CH, contralateral hippocampus; EC, entorhinal cortex; ETE, extratemporal epilepsy; ETL, extratemporal lesion; L, left; LF, lateral frontal; LT, lateral temporal; MTS, mesial temporal sclerosis; MST, multiple subpial transections; OC, orbito-frontal cortex; PH, posterior hippocampus; PI, posterior insula; R, right; RNS, responsive neurostimulation system; SEEG, stereo-electroencephalography; SFS, superior frontal sulcus; TLE, temporal lobe epilepsy; TPE, temporal plus epilepsy; UMTE, unilateral mesial temporal lobe epilepsy; UMTL, unilateral mesial temporal lobe lesion; UNTE, unilateral neocortical temporal lobe epilepsy; UNTL, unilateral neocortical temporal lesion.

**Table 4:** Brain regions involved in SEEG ictal onset zone.

Temporal Lobe Epilepsy Subtype	Patients (n = 24)	SEEG Ictal Onset, Brain Region(s) *
SOZ involved temporal cortex only	n = 17	
UMTE	7	AH, PH, A, EC
BMTE	5	AH, PH, A, EC, CH
UNTE	4	LT
UNTE **	1	AH, A, EC, LT
SOZ involved extratemporal cortex (TPE and TLE mimic)	n = 7	
U/L Lateral Temporal and Extratemporal (TPE)	3	LT, PI(2), LF+OC(1)
U/L Meslal and Lateral Temporal, and Extratemporal (TPE)	2	AH, PH, LT, LF(1), AI(1)
B/L Lateral Temporal, Extratemporal cortex and C/L hippocampus (TPE)	1	LT, PI, CH
ETE (TLE Mimic)	1	LF

A, amygdala; AH, anterior hippocampus; AI, anterior insula; B/L, bilateral; BMTE, bilateral mesial temporal lobe epilepsy; CH, contralateral hippocampus; EC, entorhinal cortex; ETE, extratemporal lobe epilepsy; LF, lateral frontal; LT, lateral temporal; OC, orbito-frontal cortex; PH, posterior hippocampus; PI, posterior insula; SEEG, stereo-electroencephalography; SOZ, seizure onset zone; TPE, temporal plus epilepsy; U/L, unilateral; UMTE, unilateral mesial temporal lobe epilepsy; UNTE, unilateral neocortical temporal lobe epilepsy. * more than one brain region could be involved in the ictal onset in the same patient; ** this patient had the left temporal pole encephalocele found after SEEG; seizure-free after the left temporal pole resection.

**Table 5:** TLE subtypes, MRI lesions, resective surgery, and surgical outcomes.

TLE Subtype	Number of Patients in Subgroup	Number of Patients Underwent Resection	MRI Lesion Related to SOZ	Engel Class Outcome	Average Follow-Up Duration, Months
UMTE	7	7	0	I-7	52
BMTE	5	3	0	I-1, III-1, IV-1	48
UNTE	5	3	1	I-3	52
ETE	1	1	0	I-1	60
TPE	6	6	2	I-3, III-2, IV-1	66

BMTE, bilateral mesial temporal lobe epilepsy; ETE, extratemporal epilepsy; SOZ, seizure onset zone; TLE, temporal lobe epilepsy; TPE, temporal plus epilepsy; UMTE, unilateral mesial temporal lobe epilepsy; UNTE, unilateral neocortical temporal lobe epilepsy.

## Data Availability

The original contributions presented in this study are included in the article. Further inquiries can be directed to the corresponding author.
